# Case of Hydrophobia

**Published:** 1859-09

**Authors:** J. Mason Warren


### SELECTIONS.

*Case of Hydrophobia.*—By J. Mason Warren, M. D

The following account was principally written from data furnished by Mr. J. Stearns, Jr., Surgical House-pupil of the Mass. General Hospital, who took much interest in investigating the facts of the case. The patient was a male child, by name Patrick Murphy, 3¼ years of age, living at 69 Endicott street, and was brought into the Hospital on June 25th, 1859.

Exactly five weeks before, he was bitten by a dog six or eight months old. The animal at the time was not thought to be rabid, although on the same day he had " snapped at and slightly bitten " a man in the hand, as was thought at the time from playfulness not unusual with puppies. The little boy had, at the time, a cracker in his hand, which the dog attempted to seize, taking into his mouth with it the whole of the right hand, and inflicting a wound on each side of the wrist. The wound on the anterior surface was from the half to two thirds of an inch in length ; that on the opposite side was like the mark from a simple puncture. The wounds at the time were treated by Dr. Owens, who cauterized them not long after the injury, and ordered a poultice. There was no further treatment of them. They were very sore for a time, particularly the one in front, but the child continued as well as usual in his general health, and nothing remarkable occurred till a week previous to his admission.

At this time the mother's attention was drawn to the patient by what she called a "dullness" coming over him, followed by a "silliness and listlessness." Four days before his entrance to the Hospital was the first onset of the paroxysms, which were described by the mother as having been quite formidable ; they were very violent when water was brought into his neighborhood, so that the mother was obliged to give up washing the child. He manifested a desire to take food and drink from his mother, though on attempting to swallow he was quite unable to effect it. For this reason he took scarcely any nourishment for four days before he was brought into the Hospital. The preceding facts were principally obtained from the parents of the child.

On his entrance into the House, he was in a highly excited condition, tossing his head and throwing about his limbs in every direction. He spit violently, or attempted to do so, as if his mouth was full of feathers or tow, occasionally crying out, or snapping at those about him, saying that he wished to bite them, and they must get out of his way. His eyes were very bright, his face pale, and there was a lividity about the eyelids and generally over the whole surface, with a quivering of the lips and muscles of the face, and constant tremor of the whole body. On taking a dose of morphine he was quieted, and the nurse prevailed on him to swallow some milk from a mug. After a time he drank a whole mug full, and ate a small piece of cake. His manner of taking the milk was not as if he had any aversion to it, but from apparent consciousness of the effort necessary to swallow. He clutched violently at the mug, with eyeballs starting out, and the whole frame undergoing the greatest agitation. The effort of swallowing was attended with a sense of suffocation, and the corners of the mouth were strongly retracted. He exhibited the same symptoms on taking cake; and from his great desire for both, appeared to be suffering much from hunger. A viscid discharge was observed to take place from the mouth, by the nurse and others. The urine was passed in great abundance through the afternoon and evening.

He became quite calm through the great attentions of the nurse, who seemed to inspire him with confidence, and went to bed with him in her arms, in spite of the remonstrances of those about her. He talked incessantly and incoherently, though at times he could be understood. He seemed to appreciate much the kindness of the nurse, and told her he should bite her, but when she put out her arm to him, he kissed and stroked it with his hand. He had several paroxysms after his entrance, with intervals of comparative quiet, the attacks being only of short duration, lasting about five minutes each. In this condition he continued most of the night, with constant watchfulness and tossing about, and at half past three, A. M., he died in one of the convulsive attacks.

No examination of the body could be obtained from the parents.

Mr. Stearns, at my request, visited the house at which the child had resided, for the purpose of obtaining some more facts in relation to the case, but did not elicit anything of importance beyond the preceding. He saw the wife of the man who was bitten on the same day with the little boy; the bite was a very slight one, on the joint of one finger, and the woman said no blood came from it. The man promised to be at the Hospital on the following day for me to examine it, but for some reason did not appear. The dog was drowned, and Mr. S. could get no farther history of it. A superstition existed with them, of which they informed him, that if the dog could heve been killed by one of the family the patient would have escaped ; also, that if the liver of the dog could have been applied to the wound, the effect would have been equally efficient, which of course naturally implied the death of the dog.

---

In connection with this case of hydrophobia, I would remark, that about twelve or fifteen years since, I proposed, at a meeting of this Society, for the purpose of obtaining information, the question, whether any case of this affection had ever occurred in Boston, or whether there was any tradition of one in the New England States; but no answer was elicited in the affirmative.

The first case reported in Boston appears to be that of Dr. Coale, in October, 1848, which was followed shortly afterward by that of Dr. Curtis, in Lowell, supposed to have been caused by the same dog, which had escaped from Boston and made his way to the latter city. This was followed by other cases in various directions, running through a course of two or three years, during which time I saw in consultation, in Brookline, a patient of Dr. Wild, and the case of a child brought into the Hospital within twelve hours after having been bitten, where the parts were freely cauterized at the time, and within twenty-four hours from the time of the accident cut out by Dr. Cabot. This patient returned home within four weeks apparently well, but by the expiration of another week the disease appeared, and she was returned to the Hospital with all the symptoms similar to those detailed above.

All these patients died after three or four days' illness, the attack coming on in an average of about five weeks from the reception of the injury.

Since that period, the contagion, if it may be so called, or inoculation, seems to have exhausted itself, and but few cases have been recorded until lately, when rumors have begun to be heard of its reappearance. I have constantly had persons call to consult me with very severe bites from dogs, but not finding from them that the animals had shown any signs of rabies, I have not thought it warrantable to apply so severe a remedy as cauterization, or excision, to an accident so common. Under the present circumstances, i. e., a disposition to rabies among the canine race, I should feel myself called upon to make a thorough application of the nitrate of silver to the wound, as recommended by Mr. Youatt, who considered this remedy as almost infallible if applied immediately, and who from his liability to be bitten always carried a bit of caustic in his pocket, and had many times made use of it with effect on his own person. Or, if circumstances required, free excision should be made of the injured part.

The following remarks of Mr. Youatt are of so much value that I have extracted them at some length: "The wound should be thoroughly washed and cleansed as soon as possible after the bite is inflicted; no sucking of the parts, as is advised by many, for the purpose of extracting the poison, as the presence of a small abrasion of the lips or interior of the mouth would most assuredly subject the parts to inoculation. If the wound be ragged, the edges may be taken off with a pair of sharp scissors; the wound must then be thoroughly cauterized with nitrate of silver (lunar caustic), being sure to introduce the caustic into the very depths of the wound, so that it will reach every particle of poison that may have insinuated itself into the flesh. If the wound is too small to admit of the stick of caustic, it may be enlarged by the knife, taking care, however, not to carry the poison into the fresh cut, which can be avoided by wiping the knife at each incision. Should the wound be made on any of the limbs, a bandage may be placed around it during the application of these remedies, the more effectually to prevent the absorption of the veins. Nitrate of silver is a most powerful neutralizer of specific poisons, and the affected parts will soon come away with the slough, no dressings being necessary, except perhaps olive oil, if there should be much inflammation of the parts. If the above plan be pursued, the patient need be under no apprehension as to the result, but make his mind perfectly easy on the point."

A question has been frequently asked, whether these symptoms might not be of a tetanic character from the irritation of the wound. There has not been the slightest appearance of trismus, or locked jaw, in any of the cases I have seen, and the lapse of time from the reception of the wound has been too long to be attributed to such a cause, the wounds having healed, and for the most part having shown little signs of irritation.

At the moment of writing this article, I have had a case of trismus or locked jaw at the Hospital, which, although not severe, affords an oppoıtunity of comparing this rare disease with hydrophobia. The patient was a woman, 45 years of age, in pretty good health, who had a large plank to fall upon her, producing a compound fracture and dislocation of the ankle-joint. I saw her about half an hour after the reception of the injury; the lower extremity of the tibia projected through a large wound at the ankle-joint, the internal malleolus being broken off and left in the wound. This I removed with a knife, so as to allow me to restore the dislocated bone to its proper place, with the hope, in the first view of the case, to save the limb. On further examination, however, when the restoration of the bone allowed of a more full investigation of the joint, I found the injury of the tibia to be complicated with a comminuted fracture of the fibula, some bits of which lay loose in the joint. Another fracture of the fibula also existed about half way up the limb. Amputation of the leg was therefore resorted to by the double flap, just above the upper fracture, in what appeared to be sound parts.

Although everything seemed to be favorable for union by the first intention, yet the wound partially suppurated, and put on a sloughy appearance, the vital powers of the tissues having probably been injured by the blow, although this at the time was not apparent. The patient, however, complained of little or no pain, but seemed to be quite comfortable and in good spirits, though with little appetite; she had no fever, and no other symptoms of constitutional irritation.

On June 30th, when I visited her in the morning, she told me that her jaws were stiff, and she could only open them about a quarter of an inch by taking hold of them with her hands. She said that she had felt some soreness in her jaws for about four days, but had not thought it of sufficient importance to mention. I at once suspected the nature of the disease, and requested Mr. Stearns to keep a close watch upon her, and inform me if anything unusual occurred; I also encouraged her to take something of a stimulating nature. She herself was not advised of our suspicions.

In the afternoon she was suddenly taken with a slight tetanic spasm, great difficulty of breathing, and coldness of the extremities; stimulants were administered, hot applications made to the feet, with other external remedies, and when I saw her about 6, P. M., she was in a very comfortable condition. Her jaws at this time had to be pried open with a bit of stick. I ordered a drachm of the solution of the sulphate of morphia to be administered every three or four hours, and as much brandy to be given as she was disposed to take.

She passed a very quiet night under the treatment directed, and on the following day pronounced herself much relieved; the stump was suppurating freely, and gave her no pain.

The mental condition of this patient and of the one with hydrophobia, it will be perceived, were strikingly different. This one was perfectly calm and collected, unlike the semi-delirious, agitated and violent state of the patient with the latter disease, the pulse not much affected, being rather below than above the natural standard; in hydrophobia it is very rapid, as has been the fact in all cases of locked jaw that I have seen. To a person who has seen the two diseases, I do not think it would be very easy to make a mistake in the diagnosis.—*Boston Medical and Surgical Journal.*

